# Role of Omega-3 Fatty Acids in the Treatment of Depressive Disorders: A Comprehensive Meta-Analysis of Randomized Clinical Trials

**DOI:** 10.1371/journal.pone.0096905

**Published:** 2014-05-07

**Authors:** Giuseppe Grosso, Andrzej Pajak, Stefano Marventano, Sabrina Castellano, Fabio Galvano, Claudio Bucolo, Filippo Drago, Filippo Caraci

**Affiliations:** 1 Department of Clinical and Molecular Biomedicine, Section of Pharmacology and Biochemistry, University of Catania, Catania, Italy; 2 Department of Epidemiology and Population Studies, Jagiellonian University Medical College, Krakow, Poland; 3 Department “G.F. Ingrassia”, Section of Hygiene and Public Health, University of Catania, Catania, Italy; 4 Department of Educational Sciences, University of Catania, Catania, Italy; 5 IRCCS Associazione Oasi Maria S.S. – Institute for Research on Mental Retardation and Brain Aging, Troina, Enna, Italy; Universidad Peruana Cayetano Heredia, Peru

## Abstract

**Background:**

Despite omega-3 polyunsaturated fatty acids (PUFA) supplementation in depressed patients have been suggested to improve depressive symptomatology, previous findings are not univocal.

**Objectives:**

To conduct an updated meta-analysis of randomized controlled trials (RCTs) of omega-3 PUFA treatment of depressive disorders, taking into account the clinical differences among patients included in the studies.

**Methods:**

A search on MEDLINE, EMBASE, PsycInfo, and the Cochrane Database of RCTs using omega-3 PUFA on patients with depressive symptoms published up to August 2013 was performed. Standardized mean difference in clinical measure of depression severity was primary outcome. Type of omega-3 used (particularly eicosapentaenoic acid [EPA] and docosahexaenoic acid [DHA]) and omega-3 as mono- or adjuvant therapy was also examined. Meta-regression analyses assessed the effects of study size, baseline depression severity, trial duration, dose of omega-3, and age of patients.

**Results:**

Meta-analysis of 11 and 8 trials conducted respectively on patients with a DSM-defined diagnosis of major depressive disorder (MDD) and patients with depressive symptomatology but no diagnosis of MDD demonstrated significant clinical benefit of omega-3 PUFA treatment compared to placebo (standardized difference in random-effects model 0.56 SD [95% CI: 0.20, 0.92] and 0.22 SD [95% CI: 0.01, 0.43], respectively; pooled analysis was 0.38 SD [95% CI: 0.18, 0.59]). Use of mainly EPA within the preparation, rather than DHA, influenced final clinical efficacy. Significant clinical efficacy had the use of omega-3 PUFA as adjuvant rather than mono-therapy. No relation between efficacy and study size, baseline depression severity, trial duration, age of patients, and study quality was found. Omega-3 PUFA resulted effective in RCTs on patients with bipolar disorder, whereas no evidence was found for those exploring their efficacy on depressive symptoms in young populations, perinatal depression, primary disease other than depression and healthy subjects.

**Conclusions:**

The use of omega-3 PUFA is effective in patients with diagnosis of MDD and on depressive patients without diagnosis of MDD.

## Introduction

Omega-3 polyunsaturated fatty acids (PUFA) eicosapentaeoic acid (EPA) and docosahexaenoic acid (DHA) have been demonstrated to be effective in cardiovascular disease (CVD) prevention due to their anti-inflammatory and cardio-protective effects [1]. Recently, new therapeutic indications for omega-3 PUFA have been proposed, such as treatment for certain forms of mental illness, including depressive disorders [2]. Indeed, some psychiatric diseases as depression may share certain pathophysiological mechanisms with CVD, namely increased production of pro-inflammatory cytokines, endothelial dysfunction, and elevations in plasma homocysteine levels [3]–[5]. The positive effects of omega-3 PUFA on depression may depend on their physiological abundant content in the human nervous system and their involvement in neurogenesis and neuroplasticity [6]. Moreover, their anti-inflammatory capacity may counteract inflammatory processes occurring in depression [7], [8]. Several ecological, cross-sectional, and prospective studies supported such hypotheses by reporting an inverse association between omega-3 intake and prevalence of depression [2]. Further clinical studies demonstrated lower concentration of omega-3 PUFA in plasma or red blood cell membranes of depressed subjects [9]–[13]. All together, these observations suggest a correlation between omega-3 PUFA and depressive disorders, justifing the rationale of a number of randomized controlled trials (RCTs) of omega-3 PUFA supplementation for the treatment of depressive disorders. The overall analysis of these studies from previous meta-analyses suggested a general benefit of omega-3 PUFA on depressive symptoms, despite certain variability in results weakened the possible validity of the findings. Indeed, results of such studies are not univocal, jeopardizing the evidence of therapeutic implications of omega-3 PUFA in depressed patients. It has been suggested that the heterogeneity between studies may depend on clinical and methodological issues, such as severity of baseline depression and methods of assessment and diagnosis of depression. Some important issues regarding therapeutic regimen have been explored in more recent meta-analysis, reporting that the positive effects of omega-3 PUFA on depressive symptoms appeared to depend more on EPA administration rather than DHA, severity of depression, and study quality [14]. However, some concerns regarding these findings still persist [15], [16]. The analyses previously conducted focused on the effects of omega-3 PUFA supplementation on depressive symptoms, but features associated with the pathophysiological nature of the depression occurring in the patients and their comorbidity status were often lacking. It is reasonable to believe that the biological effects of omega-3 PUFA may result effective in certain subtypes of depressive disorders rather than in others due to the different type of depression or clinical phenotype of the patient. Despite a full understanding of the processes leading to the depressive status is lacking, primary psychiatric disorders, such as major depression disorder (MDD) and bipolar disorders, are specific psychiatric conditions as recognized in the American Psychiatric Association’s revised fourth edition of the Diagnostic and Statistical Manual of Mental Disorders (DSM-IV) [17], marking out specific depressive symptoms that should be present as inclusion criteria to determine MDD diagnosis. The mental health examination may include the use of rating scales, such as the Hamilton Rating Scale for Depression [18], the Beck Depression Inventory [19], or the MARDS [20] for MDD, and the bipolar spectrum diagnostic scale [21] for bipolar disorders. These psychiatric diseases have indeed specific biological causes and are often known to be treated with and respond to different pharmacological interventions [22]. Another specific pathological condition is perinatal depression, which indicates the occurrence of depressive and other mood-associated symptoms during pregnancy and lactation, with a range of 5–25% of women developing post-partum depression [23]. Pregnancy and lactation are challenging periods due to a higher demand of omega-3 PUFA from the fetus and the newborn, respectively, and a low DHA status may induce depressive symptoms [24]. Despite the fact that it is not clear if the depressive status is caused by or simply precipitated by pregnancy and lactation conditions, it is however likely associated with these conditions rather than with the aforementioned causes of MDD. Similarly, psychiatric disorders occurring in young populations need special attentions because major differences between adult and juvenile depression have been well-documented, despite the reasons for such dissimilarities are not clear [25]. Actually, there is very limited evidence upon which to base conclusions about the relative effectiveness of psychological interventions or antidepressant medication, but effectiveness of these interventions cannot be fully established [26]. Finally, the occurrence of depression secondary to a different primary disease, for instance schizophrenia, Alzheimer’s disease (AD), Parkinson’s disease, and CVD, may raise doubts on the pathophysiological mechanisms that cause the depressive symptomatology. In addition, despite it is of interested to examine the role of omega-3 PUFA on potential mood depression in healthy subjects, it is important to underline that preventive and therapeutic pathways may differ each other. Thus, altogether, the choice in previous meta-analyses to pool together studies with such different baseline conditions, in which depression occurred, may have affected the quality of the studies as well as utilizability of the results [27], [28]. Moreover, the last meta-analysis included studies up to 2010 [29]. Thus, the aim of this study was to update the current knowledge about the overall clinical efficacy of omega-3 fatty acids (particularly EPA and DHA) in previous and more recent RCTs published in the last years, minimizing, from a clinical point of view, the differences among the populations of patients included in the studies, finally focusing on patients with a DSM-defined diagnosis of MDD.

## Methods

**Figure 1:**
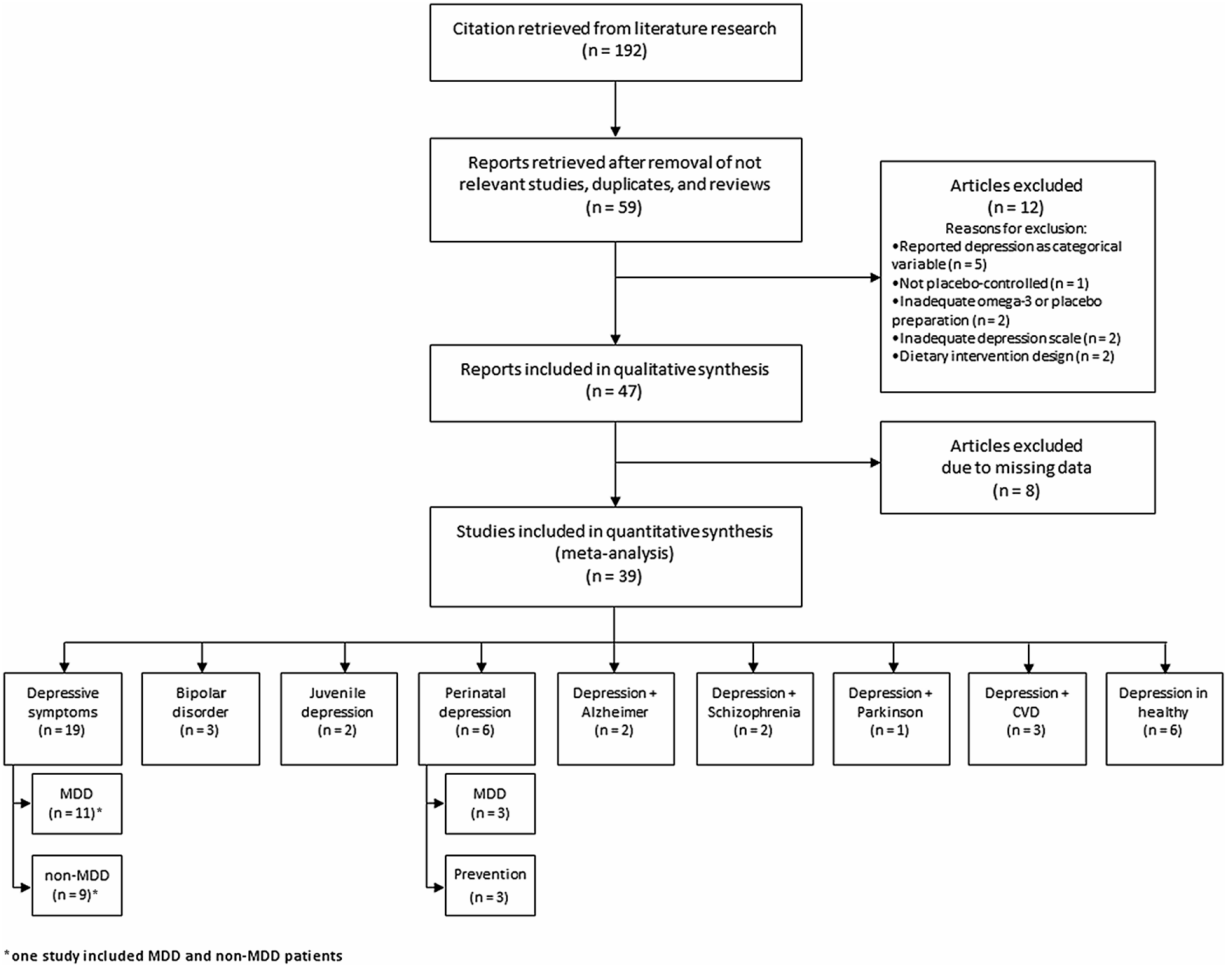
Process of inclusion of trials for systematic review and meta-analysis of studies on omega-3 fatty acids and depressive symptoms.

A comprehensive search on MEDLINE, EMBASE, PsycInfo, and the Cochrane Database systematic Reviews of all RCTs using omega-3 PUFA on patients with depressive symptoms published up to August 2013 was performed. Articles of potential interest were identified by using the following search terms: “omega-3”, “polyunsaturated fatty acids”, “PUFA”, “trial”, “EPA”, “DHA”, combined with the following terms: “depression”, “depressive disorder”, “depressed mood”, “bipolar”, combined with “perinatal”, “post-partum”, “CVD”, “schizophrenia”, “Parkinson”, “Alzheimer”, “diabetes”, “angina”. Among the 192 articles retrieved, RCTs were identified and screened by reading the abstract and, when necessary, the full text, in order to select those articles relevant for the analysis. The reference list of the relevant reports was also inspected to identify any additional trials not previously identified. The process of identification and inclusion of trials is summarized in Figure 1. Inclusion criteria were the following: (i) studies conducted on humans; (ii) randomized design; (iii) placebo controlled; (iv) use of omega-3 PUFA supplement which relative amount could be quantified; (v) exploring changes in depressive symptoms as primary or secondary outcome. Exclusion criteria were the following: (i) studies reporting insufficient statistics or results; (ii) adopted a dietary intervention design. Study quality was measured in a 13-point scale including the Jadad criteria [30] and specific information regarding (i) registration of RCT before conducting the study, (ii) adequate blinding of the researchers, (iii) the use of an intention-to-treat analysis, (iv) control for patients’ diet (i.e., number of servings of fish), (v) assessment of compliance through measurement of plasma fatty acids, (vi) significant differences at baseline, (vii) adequate sample calculation, whether (viii) depression was the primary outcome, and (ix) number and reasons of withdrawal were mentioned. Data were abstracted independently from each identified trial by GG and SM using a standard data abstraction form. This process was independently performed by two researchers and discordances were discussed and risolved.

Out of 59 originally selected studies, one [31] was excluded because of having a non-randomize non-placebo controlled design; two [32], [33], because there was used a dietary intervention design; five [34]–[38], because the depressive status was reported as a categorical variable rather than a rating scale; two [39], [40], because an inadequate or poorly comparable rating score of depression was used; two [41], [42], because poorly comparable omega-3 PUFA or placebo preparations. This selection strategy resulted in a final selection of 47 studies eligible to be included in the present systematic review.

The clinical outcome of interest was the standardized mean difference in the change from baseline to endpoint scores on a depression rating scale, in patients taking omega-3 PUFA supplements *vs.* patients taking placebo. Preferred rating scales for measuring depression severity were the Hamilton Depression Rating Scale (HDRS), either the 9-item short form, 17-item, 21-item or 25-items scales, and the Montgomery Asberg Depression Rating Scale (MADRS) [20], [43], [44]. When available, HDRS scores from each study were used. If the HDRS was not available we used the MADRS. If neither HDRS nor MADRS data were available, we used the clinician rated measure of depression that the investigators identified as their primary outcome.

Among selected RCTs lacking in data, such as means and/or standard deviations (SDs), the data of one study [45] were provided by authors; SDs and 95% confidence intervals (CIs) of five studies [46]–[50] were retrieved from graphs; data of one study [51] were medians; and data of three studies [52]–[54] were imputed from data from all other trials using the same measure for depression as described elsewhere [55]. Eight studies [56]–[63] were finally excluded from the meta-analysis due to lacking data, resulting in a total number of 39 studies to be included in the analysis.

Effects due to participant diagnosis were investigated by grouping studies according to the most relevant clinical characteristics of the population on which they were conducted, as follows: (i) Depressed patients (including DSM-defined diagnosis of MDD and general assessment of depression without clinical visit); (ii) Bipolar disorder patients (including bipolar disorder during pregnancy); (iii) Children or adolescents with depression or bipolar disorder; (iv) Women with perinatal depression (including DSM-defined diagnosis of MDD and prevention of post-partum depression); (v) Mild-cognitive impairment or AD patients; (vi) Schizophrenic patients; (vii) Parkinson’s disease patients; (viii) Patients with concomitant CVDs; and (ix) Healthy subjects.

Data regarding type of diagnosis, number of subjects enrolled in the trial, on-going therapy, (TRATT.) type of supplement used in the intervention, type of placebo, daily dose, duration of the intervention, outcome measures, and information to retrieve the study quality were collected. Those RCTs reporting more than one dose of omega-3 PUFA [54], [64]–[68] or more than one formulation (i.e., EPA or DHA separately) [48], [51], [69], were considered as separate studies in the pooled analyses. One study [70] enrolled different populations (MDD and non-MDD patients), thus each population was also included in the meta-analysis as a separate study.

### Statistical Analysis

Continuous data were reported as mean and SDs and listed in descriptive tables. All depression scales’ means and SDs at baseline and end of follow-up period of both intervention and control groups were combined [71] and the standardized mean effect for all trials was calculated by using Hedges adjusted *g* in order to correct for small sample bias [72]. Both random- and fixed-effects models were used to estimate the overall effect size. Heterogeneity was investigated by using Higgins’ *I^2^* statistic [73], [74]. When heterogeneity between results of the studies exists, the random-effect models were preferred.

Possible publication bias for the analysis regarding RCTs conducted on MDD patients (MDD group, n = 11) and those not diagnosed with DSM-IV criteria (non-MDD group, n = 9) was investigated by drawing a funnel plot to look for funnel plot asymmetry [71] and meta-regression based on study size. Meta-regression was performed using linear regression, with the effect size (SMD) of trials as the dependent variable and the variables of interest as the independent variable. The generic inverse variance method was used to weight trials. Effects due to severity of depressive symptoms, age of patients, and study quality were also investigated by using meta-regression based on standardized baseline depression scores, mean age of the study participants, and our modified Jadad scores of the studies, respectively. The effects of trial duration, EPA and DHA dose in omega-3 preparations, and the use as mono or adjuvant therapy were also examined. Particularly, the qualitative analysis of the type of supplementation used was investigated grouping the studies in those using mainly EPA (EPA >50% of the dose) and mainly DHA (DHA >50% of the dose). A further analysis was computed by splitting the grouping in mainly EPA, pure EPA, mainly DHA, and pure DHA supplementation. As well, the therapeutic approach was investigated by grouping studies using omega-3 in monotherapy or as adjuvant therapy together with antidepressant drugs. The quantitative analysis of the dose was computed by a meta-regression analysis of the EPA and DHA doses used.

Random- and fixed-effects models, forest and funnel plots, and Higgins’ *I^2^* statistics were performed in Review Manager (RevMan) version 5.2 (Copenhagen: The Nordic Cochrane Centre, The Cochrane Collaboration), meta-regression analyses were performed in SPSS version 17 (SPSS Inc., Chicago, IL, USA).

## Results

### Overall Studies

The most relevant features of the 47 studies included in this systematic review and meta-analysis are displayed in Table 1. Considerable differences among studies were found for all characteristics examined. The average quality of the studies was about 9 over a maximum score of 13 (range 5–13). The mean length of the trials was about 16 weeks (range 4–160), 36 studies used a mixed intervention with EPA+DHA, 14 pure EPA and 4 pure DHA. The average dose of EPA+DHA was 1.39 g (range 0.63–6.2 of EPA and 0.27–3.4 of DHA), whereas 1.93 g (range 1–6) and 0.86 g (range 0.22–2) were the average doses of pure EPA and DHA, respectively (Table 1). The most of RCTs used the Hamilton Depression Rating Scale [46], [48], [49], [54], [57], [58], [63], [65], [69], [75]–[84], 10 studies [46], [49], [56], [68], [79], [85]–[89] used the Beck Depression Inventory, and 13 studies [47], [50], [54], [62], [64], [67], [77], [84], [90]–[94] the Montgomery-Asberg Depression Scale as the main outcome measure. Among the studies not included in the quantitative analysis,due to lack of data, one was conducted on patients with obsessive-compulsive disorder [57] and one on patients with chronic fatigue syndrome [56], both reporting no relevant effects of omega-3 fatty acids compared with placebo; four studies conducted in bipolar depressed patients [58], [59], [61], [63] reporting that there were no significant differences on any outcome measure between the EPA and placebo groups; one study on diabetes mellitus patients with MDD [62] reporting no effect of omega-3 fatty acids on depression severity; and one on older adults with mild cognitive impairment suggesting that increased intakes of DHA and EPA can reduce depressive symptoms and the risk of progressing to dementia [60].

**Table 1:** Randomized controlled trials investigating effects of omega-3 polyunsaturated fatty acids (PUFAs) on depressed mood listed in chronological order by type of depressive disorder.

Author	Year	Partecipating Group	Subjects, n (I/C)	*Type of treatment*	Intervention	Placebo	Daily dose	Duration (weeks)	Outcome measure	Study quality
*MDD*										
Nemets [76]	2002	Pz with MDD	20 (10/10)	All but 1 used antidepressant	E-EPA	NR	2 g	4	HDRS	8
Marangell [77]	2003	Pz with MDD	36 (18/18)	None	DHA	NR	2 g	6	MADRSHDRS	7
Su [78]	2003	Pz with MDD	28 (14/14)	Mixed antidepressants	EPA+DHA	Olive oil ethyl esters	4.4 g EPA+2.2 g DHA	8	HDRS	8
Grenyer [46]	2007	Pz with MDD	83 (40/43)	Mixed antidepressants	EPA+DHA	Olive oil	0.6 g EPA+2.2 g DHA	16	BDI, HDRS	9
Jazayeri [48]	2008	Pz with MDD	60 (20/20/20)	Fluoxetine	E-EPA, E-EPA+fluoxetine	Rapeseed oil	1.0 g E-EPA	8	HDRS	10
Mischoulon [83]	2009	Pz with MDD	57 (28/29)	Psychotherapy	EPA (+0.2% dl-alpha-tocopherol)	Paraffin oil and 0.2% dl-alpha-tocopherol	1 g EPA	8	HDRS −17	11
Rondanelli [95]	2010	Pz with MDD (only women >66)	46 (22/24)	None	EPA+DHA	Paraffin oil	1.67 g EPA+0.83 DHA	8	GDS, SF-36	10
Rondanelli [53]	2011	Pz with MDD (only women >66)	46 (22/24)	None	EPA+DHA	Paraffin oil	1.67 g EPA+0.83 DHA	8	GDS, SF-36	10
Gertsik [84]	2012	Pz with MDD	42 (21/21)	Citalopram	EPA+DHA	Olive oil	0.9 g EPA+0.2 g DHA	8	HDRS, BDI, MADRS, CGI	7
Rizzo [96]	2012	Pz with MDD (only women >66)	46 (22/24)	NR	EPA+DHA	Paraffin oil,	2.5 g of n-3 PUFA with EPA/DHA 2∶1	8	GDS	8
*Non-MDD*										
Behan [39]	1990	Pz with post viral fatigue	63 (39/24)	NR	EPA+DHA	Liquid paraffin+0.4 g LA	0.14 g EPA+0.09 g DHA	13	4-point Linkert scale	8
Warren [56]	1999	Pz with chronic fatigue syndrome	50 (24/26)	None	EPA+DHA	Sunflower oil	0.14 g EPA+0.9 g DHA	13	BDI	8
Peet [54]	2002	Pz treated for depression	70 (17/18/17/18)	Mixed antidepressants	E-EPA	Liquid paraffin	1 g; 2 g; 4 g	12	HDRS, MADRS, BDI	7
Zanarini [91]	2003	Pz with borderline personality disorder	30 (20/10)	Heterogeneous	E-EPA	Mineral oil	1 g	8	MADRS	6
Fux [57]	2004	Pz with obsessive compulsive disorder	11 (11/11) (within- subjects crossover design)	Heterogeneous	E-EPA	Liquid paraffin	2 g	6	HDRS	6
Silvers [79]	2005	Pz treated for depression	77 (40/37)	Mixed antidepressants	EPA+DHA	Olive oil	0.6 g EPA+2.4 g DHA	12	HDRS-SF, BDI	10
Hallahan [49]	2007	Pz with recurrent self-harm	49 (22/27)	Mixed antidepressants	EPA+DHA	Corn oil+1% n23 PUFAs	1.2 g EPA+0.9 DHA	12	BDI, HDRS	9
Rogers [86]	2008	Untreated pz with mild-to-moderate depression	218 (109/109)	None	EPA+DHA	Olive oil	0.63 EPA 0.85 DHA	12	DASS, BDI, GHQ, Mood Diary	12
Lucas [70]	2009	Pz with psychological distress	120 (59/61)	None	EPA +DHA(ethyl esters)	Sunflower oil	1.05 g EPA.0.15 g DHA	8	PGWB, HDRS, CGI, HSCL-D-20	12
Tajalizadekhoob [97]	2011	Pz with mild-to-moderate depression (>66 yrs)	66 (33/33)	55 mixed antidepressants, 11 none	EPA+DHA	Coconut oil	0.180 g EPA+0.120 g DHA	24	GDS-15	10
Antypa [89]	2012	Pz with a history of at least one major depressive episode	71 (36/35)	7 mixed antidepressants, 6 heterogeneous, 58 none	EPA+DHA	Olive oil	1.74 g EPA+0.25 g DHA	4	BDI-II	9
Mozaffari-Khosravi [69]	2013	Pz with mild-to-moderate depression	81 (27/27/27)	Mixed antidepressants	EPA or DHA	Coconut oil	1 g EPA or 1 g DHA	12	HDRS-17	12
Sohrabi [40]	2013	Pz with pre-mestrual syndrome	139 (70/69)	113 sedative, 26 none	EPA+DHA	NR	0.24 EPA +0.36 DHA	12	VAS	8
*Bipolar disorder*										
Stoll [75]	1999	Pz with bipolar disorder	30 (14/16)	Heterogeneous	EPA+DHA	Olive oil	6.2 g EPA+3.4 g DHA	16	HDRS	8
Hirashima [58]	2004	Pz with bipolar disorder	21 (12/9)	Heterogeneous	EPA+DHA	NR	5–5.2 g EPA+3–3.4 g DHA or 1.3 g EPA+0.7 g DHA	4	HDRS	4
Chiu [63]	2005	Pz with bipolar disorder	15 (NR)	Lorazepam,valproate	EPA+DHA	Olive oil	0.44 g EPA+0.24 g DHA	4	HDRS	5
Frangou [65]	2006	Pz with bipolar disorder	75 (24/25/26)	Heterogeneous	E-EPA	Paraffin oil	1 g; 2 g	12	HDRS	9
Keck [59]	2006	Pz with bipolar disorder	116 (59/57)	Mood stabilizing	E-EPA	Liquid paraffin	6 g	17	IDS-C	9
Frangou [80]	2007	Pz with bipolar disorder	14 (7/7)	Lithium	E-EPA	Liquidparaffin	2 g E-EPA	12	HDRS	8
*Depression or bipolar disorder in children and adolescents*										
Nemets [98]	2006	Children with MDD	28 (13/15)	5 Methylphenidate	EPA+DHA	Olive oil or safflower oil	0.38–0.40 g EPA. 0.18–0.20 g DHA	16	CDRS, CDI, CGI	7
Gracious [61]	2010	Children and adolescents with bipolar disorder	51 (NR)	lithium, atypical antipsychotic	α-LNA	Olive oil	0.55–6.6 α-LNA	16	CDRS-R, CPRS, CGI-BP	11
Amminger [92]	2010	Adolescents at risk of psycosis	81 (41/40)	Heterogeneous	EPA+DHA	Coconut oil	0.70 g EPA+0.48 g DHA	48	MADRS, SCID	11
*Peritanal MDD*										
Freeman [99]	2008	Pz with MDD during pregnancy	59 (31/28)	Psychotherapy	EPA+DHA	Corn oil+1% fish oil	1.1 g EPA+0.8 g DHA	8	EPDS, HDRS, CGI	8
Su [82]	2008	Pz with MDD during pregnancy	36 (18/18)	None	EPA+DHA	Olive oil ethyl esters	2.2 g EPA 1.2 g DHA	8	HDRS, EPDS, BDI-21	10
Rees [93]	2008	Pz with MDD during pregnancy	26 (13/13)	None	EPA+DHA	Sunola oil	0.42 g EPA. 1.64 g DHA	6	EPDS, HDRS, MADRS	11
*Prevention of post-partum depression*										
Llorente [85]	2003	Healthy pregnant women	99 (44/45)	None	DHA	NR	0.2 g	16	BDI	10
Doornbos [51]	2009	Healthy pregnant women	119 (42/41/36)	Unclear	DHA DHA+AA	Soybean oil	0.22 g DHA. 0.22 g DHA. 0.22 g AA	28	EPDS (Dutch), PPBQ	5
Mozurkewich [68]	2013	Healthy pregnant women	126	Unclear	EPA+DHA	soy oil	1.06 g EPA+0.27 DHA or 0.9 DHA+0.18 EPA		BDI	12
*Depressive symptoms in pz with Alzheimer disease or mild cognitive impairment*										
Chiu [81]	2008	Pz with Alzheimer disease or mild cognitive impairment	46 (24/22)	Unclear	EPA+DHA	Olive oil ethyl esters	1.08 g EPA+0.72 g DHA	24	MMSE, HDRS	9
Freund-Levi [50]	2008	Pz with Alzheimer disease	204 (103/101)	Various	EPA+DHA	Corn oil+0.6 g LA	0.6 g EPA. 1.72 g DHA	26	MADRS, NPI	8
Sinn [60]	2012	Pz with mild cognitive impairment (>65)	50 (17/18/15)	Unclear	EPA+DHA	LA 2,2 g	1.67 g EPA+0.16 g DHA or 1.55 g DHA+0.40 g EPA	24	GDS	9
*Depressive symptoms in pz with schizophrenia*										
Fenton [90]	2001	Pz with schizophrenia	87 (43/44)	All but 1 used neuroleptic	E-EPA	Mineral oil	3 g	16	MADRS	10
Peet [64]	2002	Pz with schizophrenia	115 (29/28/27/31)	31 clozapine, 48 atypical antipsychotics, 36 typical psychotic	E-EPA	Liquid paraffin	1 g; 2 g; 4 g	12	MADRS	9
*MDD in pz with Parkinson’s disease*										
Da Silva [47]	2008	Pz with Parkinson’s disease and MDD	29 [NAD: 13 (6/7) AD: 16 (8/8)]	26 levodopa, 19 pramipexol, 5 amantadine, 4 COMT inhibitors, 6 SSRI, 4 tricyclics, 2 trazodone	EPA+DHA	Mineral oil	0.72 g EPA. 0.48 g DHA	12	MADRS, BDI, CGI	10
*Depressive symptoms in pz with CVD*										
Carney [87]	2009	Pz with coronary heart disease and MDD	122 (62/60)	sertraline 50 mg/day	EPA+DHA	Corn oil	0.93 g EPA; 0.75 g DHA	10	BDI-II, HDRS-17	10
Bot [94]	2010	Pz with diabetes mellitus and MDD	25 (13/12)	antidepressant medication	EPA	Rapeseed oil and medium chain triglycerides	1 g	12	MADRS	12
Giltay [100]	2011	Pz post myocardial infarction	4116	antidepressant medication	EPA+DHA		0.4 EPA-DHA/d. 2 ALA/d. 0.4 EPA-DHA+2 ALA	160	GDS, LOT-R	10
Bot [62]	2011	Pz with diabetes mellitus and MDD	25 (13/12)	antidepressant medication	EPA	Rapeseed oil and medium chaintriglycerides	1 g	12	MADRS	10
Andreeva [37]	2012	Pz CVD survivors	2501 (620/633/622/626)	Antidepressant used by 130 (63/67)	B vitamins and n3 fatty acids (EPA+DHA), n3 fatty acids, B vitamins	B vitamins	600 mg EPA and DHA in a 2∶1 ratio	52	GDS	9
*Depressive symptoms in healthy subjects*										
Fontani [52]	2005	Healthy subjects	33 (cross-over design)	None	EPA+DHA	Olive oil	1·60 g EPA+0·80 g DHA+0·40 g other omega-3 fatty acids	5	POMS	7
Van de Rest [68]	2008	Healthy subjects	302 (96/100/106)	Unclear	EPA+DHA	Sunflower oil	High: 1.093 g EPA. 0.847 g DHA; Low: 0.226 g EPA. 0.176 g DHA	26	CES-D, MADRS, GDS-15	12
Antypa [88]	2009	Healthy subjects	(56;>27/>27)	None	EPA+DHA	Olive oil	1.74 g EPA, 0.25 g DHA	4	MINI, BDI-II, POMS, LEIDS-R	9
Kiecolt-Glaser [102]	2011	Healthy subjects	68 (34/34)	None	EPA+DHA	Palm, olive, soy, canola, and coco butter oils	2.085 g EPA 0.348 g DHA	12	CES-D	11
DeFina [101]	2011	Healthy subjects (overweight)	128 (64/64)	None	EPA+DHA	Soybean and corn oils	3.0 g EPA and DHA in a 5∶1 ratio (5 g EPA 1 g DHA)	24	POMS	8
Kiecolt-Glaser [66]	2012	Healthy subjects (overweight)	138 (46/46/46)	None	EPA+DHA	Palm, olive, soy, canola, and coco butter oils	2.09 g EPA+0.35 g DHA; n3 1.25 g middle group	16	CES-D	13

AD: anti-depression; BDI: Beck Depression Inventory; CES-D: Center for Epidemiological Studies Depression Scale; CDRS: Children Depression Rating Scale; CPRS: Comprehensive Psychopathological Rating Scale; GDS: Geriatric Depression Scale; CGI: Clinical Global Impression; CGI-BP: Clinical Global Impression Bipolar; DHA: docosahexaenoic acid; E-EPA: etyl-eicosapentaenoic acid; EPDS: Edinburgh Postnatal Depression Scale; HDRS: Hamilton Depression Rating Scale; I/C: intervention/control; IDS-C: Inventory of Depressive Symptomatology Clinician; LEIDS-R: Leiden Index of Depression Severity Revised; LOT-R: Revised Life Orientation Test; MADRS: Montgomery Åsberg Depression Rating Scale; MINI: Mini International Neuropsychiatric Interview; MMSE: Mini-Mental State Evaluation; NAD: non anti-depression; NPI: Neuropsychiatric Inventory; POMS: Profile of Mood States; PPBQ: Papolos Pediatric Bipolar Questionnaire; SCID: Structural Clinical Interview for Depression; VAS: Visual Analog Score.

### Depression (MDD and Non-MDD Groups)

**Figure 2:**
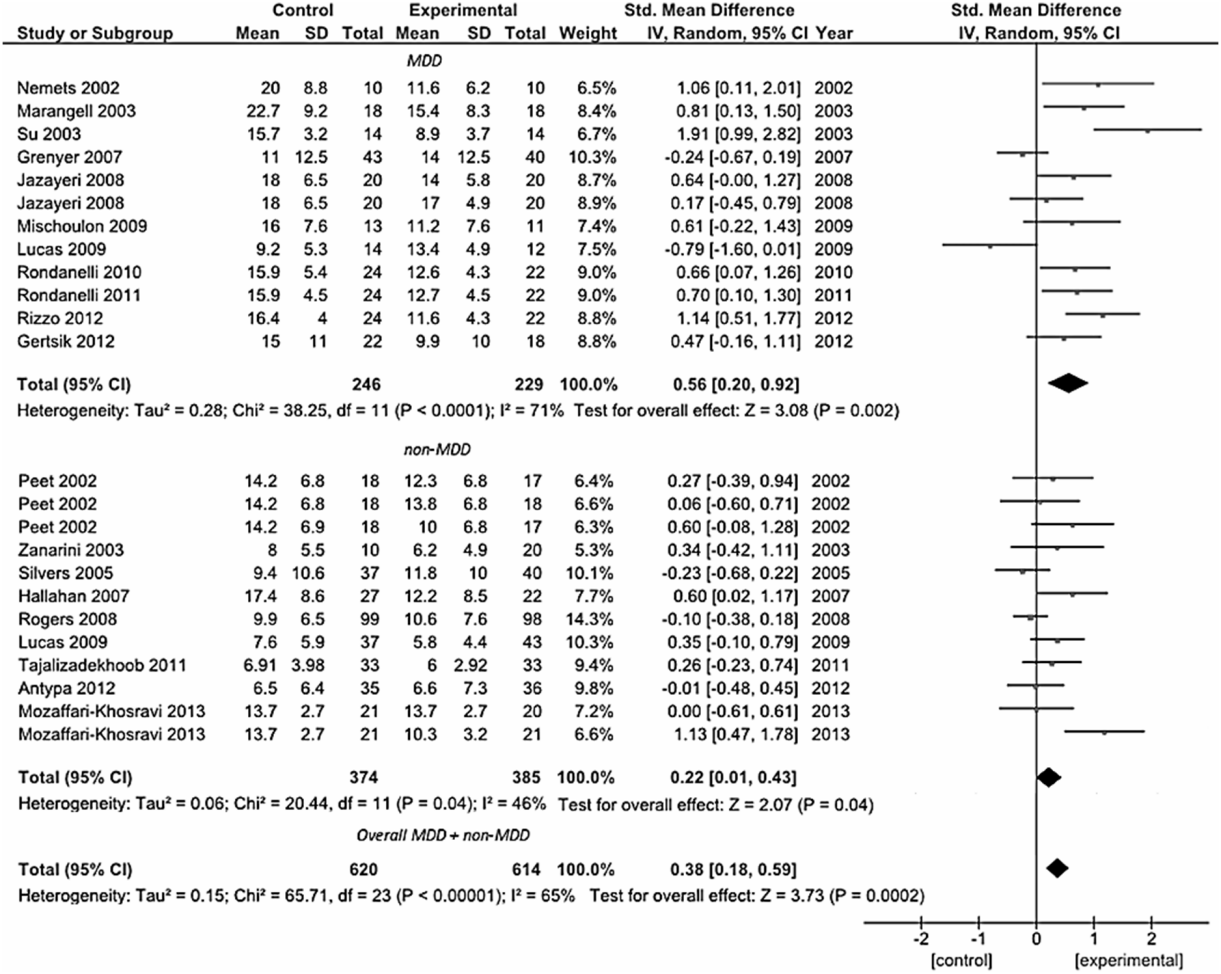
Forest plot showing individual and combined effect size estimates and 95% CIs for 19 trials grouped in those conducted on patients with a DSM-defined diagnosis of major depressive disorder (MDD group, n = 11) and those on patients with an assessment of depression but not rigorously diagnosed according to the DSM criteria (non-MDD group, n = 8). Black squares: indicate the weighting given to the trial in the overall pooled estimate; lines: indicate the 95% CIs; rhombus: indicate the combined effect size.

A total of 19 studies were included in the first pooled analysis conducted in patients with depressive symptoms (Figure 2). Among them, 11 trials were conducted in patients with a DSM-defined diagnosis of MDD, including 8 studies conducted in adults [46], [48], [70], [76]–[78], [83], [84] and 3 studies in elderly patients [53], [95], [96]. The pooled standardized difference in means using a fixed-effects model for the MDD group was 0.47 SD (95% CI: 0.29, 0.66), which suggests a beneficial effect of omega-3 fatty acids on depressed mood compared with placebo in patients with diagnosis of MDD. The pooled standardized difference in means in a random-effects model was 0.56 SD (95% CI: 0.20, 0.92). The remaining 8 were those conducted on patients with an assessment of depression but not rigorously diagnosed according to the DSM criteria, and included patients with depressive symptoms despite on-going treatment [54], [69], [79], [97], women with borderline personality disorder [91], patients with recurrent self-harm [49], people with mild to severe depressed mood not taking medications [86], post-menopausal women with psychological distress and depressive symptoms [70], and subjects with a history of at least one major depressive episode [89], whereas two studies were excluded due to lack of data [56], [57]. Despite patients pooled in this analysis were not homogeneous in terms of health status, all studies clearly reported to have included subjects with no other psychiatric or neurological illnesses such as AD, Parkinson’s disease, as well as no history of any end-stage diseases, CVDs, or any unstable medical conditions, thus to make them comparable each other for our purposes. Similar results were found for this group of patients (standardized mean difference – fixed effects-model: 0.15 SD, 95% CI: 0.01, 0.30; random-effects model: 0.22 SD, 95% CI: 0.01, 0.43). For both MDD and non-MDD groups, there was evidence of heterogeneity (MDD group, *I^2^* = 71%, *P*<0.001; non-MDD group, *I^2^* = 46%, *P* = 0.04).

**Figure 3:**
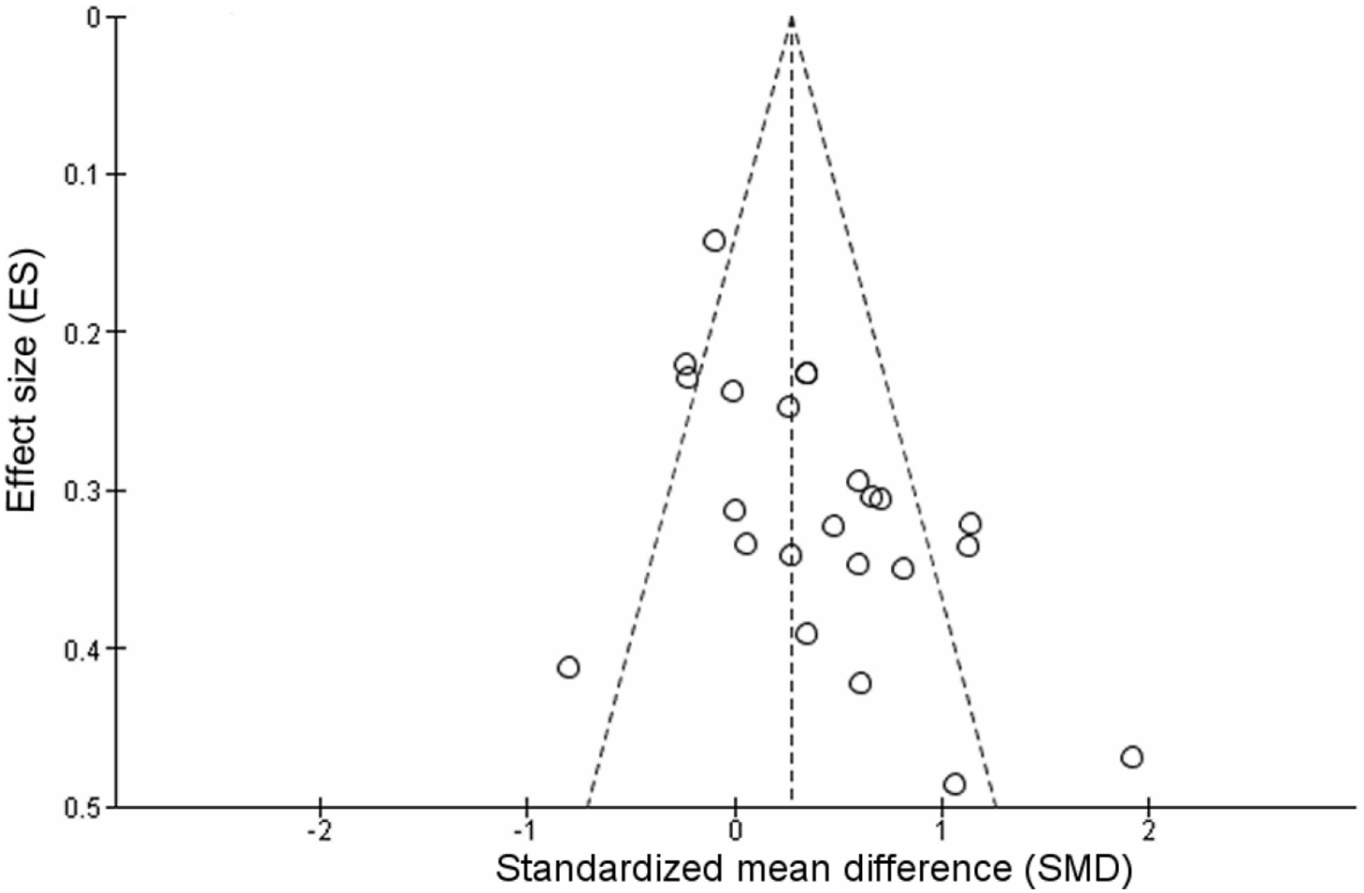
Funnel plot of effect size estimates for individual trials conducted on patients with depressive disorder without secondary comordibities (MDD group and non-MDD group, n = 19).

The overall analysis including both groups was conducted to assess whether results were different considering a mood-improving effect on depressive symptoms in patients with non-organic, metabolic, nor genetic-related neurodegenerative disease. The pooled standardized difference in means using a fixed-effects model was 0.27 SD (95% CI: 0.16, 0.39), and the pooled standardized difference in means using a random-effects model was 0.38 SD (95% CI: 0.18, 0.59). However, there was evidence of heterogeneity (*I^2^* = 65%, *P*<0.001). To test this heterogeneity, a funnel plot was drawn and is shown in Figure 3.The funnel plot did not show considerable evidence of asymmetry. Meta-regression of study effect size, based on study size, did not present significant association (regression coefficient  =  −0.108, 95% CI: −0.224, 0.012; *P* = 0.066) indicating no role of the sample size in determining the results of the analysis.

A meta-regression analysis was performed of standardized mean depression scores on baseline depression scores to test whether the gravity of depression at baseline may play a role in the efficacy of omega-3 fatty supplementation. The analysis showed no relation between baseline depression scores and efficacy for all studies (regression coefficient  = 0.019, 95% CI: −0.009, 0.047; *P* = 0.167) as well as for MDD patients (regression coefficient  = 0.008, 95% CI: −0.053, 0.068; *P* = 0.787) and non-MDD (regression coefficient  = 0.019, 95% CI: −0.017, 0.054; *P* = 0.270) separately. Even taking into account the comparison of studies using the same depression scale (HDRS), no significant relation between baseline depression scores and efficacy was found (data not shown).

**Figure 4:**
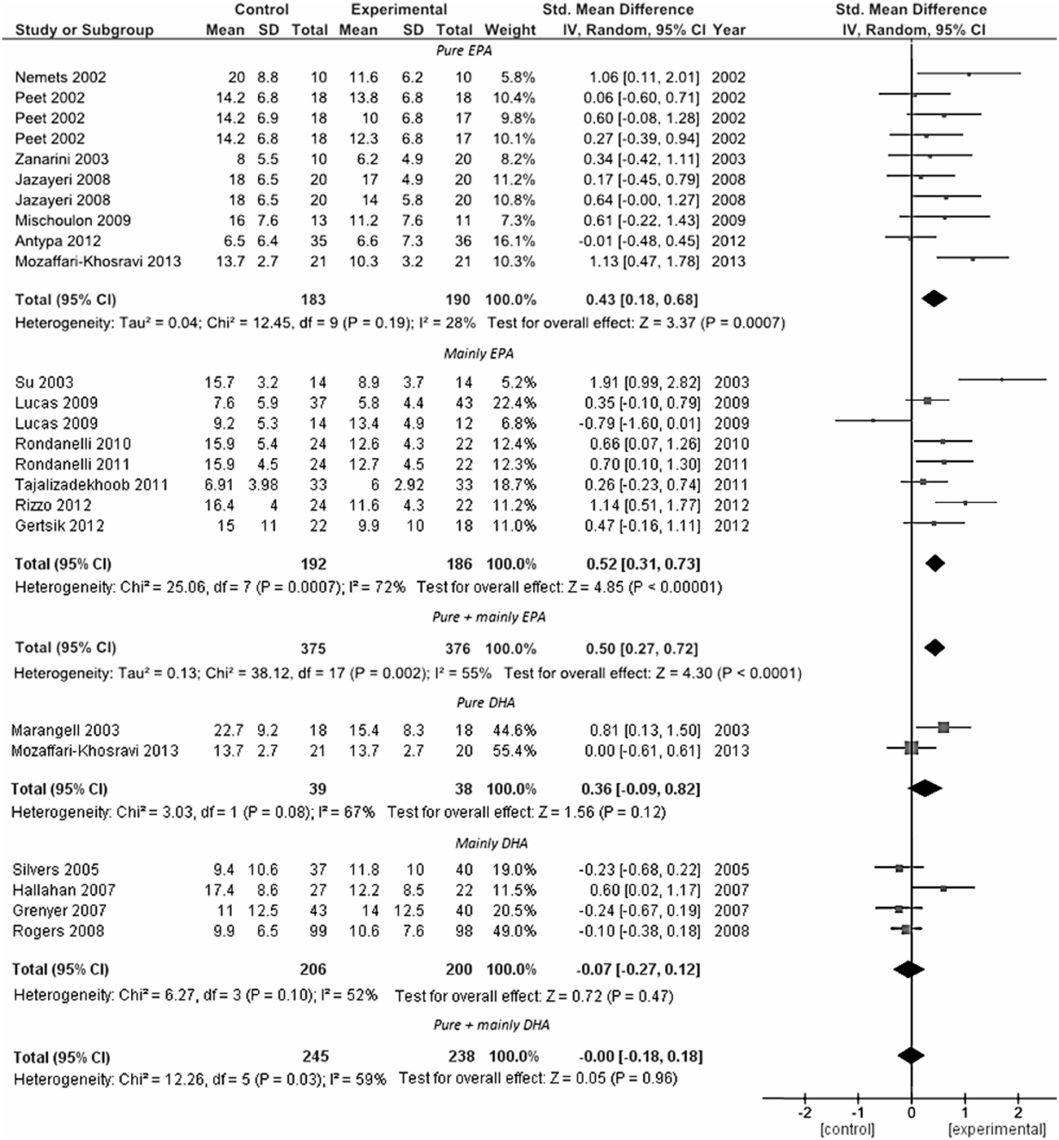
Forest plot examining the effect of the type of omega-3 PUFA supplementation employed on the reduction in depressive symptoms (MDD group and non-MDD group, n = 19).

Analysis conducted to explore the role of type (namely, the administration of mainly EPA or DHA supplementation) and dose (separately for EPA and DHA) of omega-3 supplement used showed that the use of mainly EPA within the preparation, rather than DHA, appeared to influence final clinical efficacy (standardized mean difference – fixed effects-model: 0.46 SD, 95% CI: 0.31, 0.61; random-effects model: 0.50 SD, 95% CI: 0.27, 0.72) (Figure 4). Despite heterogeneity fallen by 55%, it remained significantly high (*P* = 0.002). When the analysis was split in mainly EPA, pure EPA, mainly DHA, and pure DHA supplementation, both the EPA preparations were significant (for pure EPA, standardized mean difference – fixed effects-model: 0.40 SD, 95% CI: 0.19, 0.61; random-effects model: 0.43 SD, 95% CI: 0.18, 0.68) and the heterogeneity fallen to 28% (*P* = 0.19). This result indicates that despite the overall heterogeneity represented an underlying true difference in effect sizes across studies, it may be strongly affected by type of formulation of omega-3 fatty acids used.

The meta-regression analyses exploring the role of the dose of omega-3 fatty acids revealed that the total dose of DHA were unrelated to efficacy (regression coefficient  =  −0.066, 95% CI: −0.471, 0.603; *P* = 0.801), whereas the dose of EPA formulation resulted related to efficacy both for all MDD plus non-MDD patients (regression coefficient  = 0.477, 95% CI: 0.084, 0.869; *P* = 0.02). However, when the analyses was repeated separately for each group, the association remained significant only for MDD patients (regression coefficient  = 0.746, 95% CI: 0.100, 1.392; *P* = 0.028) whereas lost significance for non-MDD patients (regression coefficient  = 0.215, 95% CI: −0.288, 0.718; *P* = 0.359).

No relation between study size (regression coefficient  =  −0.109, 95% CI: −0.231, 0.012; *P* = 0.075, baseline depression severity (regression coefficient  = 0.026, 95% CI: −0.007, 0.060; *P* = 0.116), trial duration (regression coefficient  =  −0.058, 95% CI: −0.153, 0.038; *P* = 0.223), age of patients (regression coefficient  = 0.013, 95% CI: −0.10, 0.036; *P* = 0.879), and study quality (regression coefficient  =  −0.142, 95% CI: −0.357, 0.072; *P* = 0.183) and omega-3 PUFA efficacy was found, despite study quality almost reached significance when considered only for RCTs conducted on patients with MDD (regression coefficient  =  −0.403, 95% CI: −0.857, 0.052; *P* = 0.077). On the contrary, fixed- and random-effect models of RCTs grouped by use of omega-3 PUFA as mono- or adjuvant therapy revealed a significant effect when they were used in combination with standard antidepressant therapy (standardized mean difference – fixed effects-model: 0.26 SD, 95% CI: 0.09, 0.44; random-effects model: 0.39 SD, 95% CI: 0.06, 0.71).

### Bipolar Disorder

**Figure 5:**
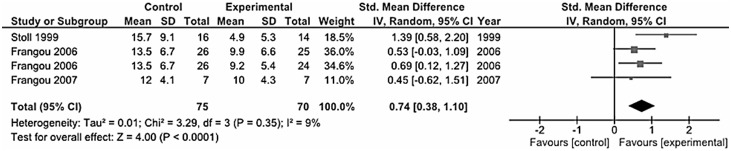
Forest plot showing individual and combined effect size estimates and 95% CIs for 3 trials conducted on patients with bipolar depression.

In our systematic review we collected 7 trials conducted on patients with bipolar disorder (both type I and II) [58], [59], [61], [63], [65], [75], [80] (Table 1). The only three studies pooled for the analysis included one study [65] that accounted for more than 70% of the weight of the analysis, that together with others [75], [80] resulted in a significant effect of omega-3 fatty acids in ameliorating depressive symptoms in adults with bipolar disorder (standardized mean difference – fixed effects-model: 0.73 SD, 95% CI: 0.39, 1.07; random-effects model: 0.74 SD, 95% CI: 0.38, 1.10; *I^2^* = 9%, *P* = 0.35) (Figure 5).

### Depression or Bipolar Disorder in Children and Adolescents

Among the studies conducted on depression occurring in youth, one study [98] documented a positive effect of omega-3 fatty acids in improving the mood of children diagnosed of MDD and one study conducted on adolescents at high risk of psychosis [92] reported that omega-3 fatty acids significantly reduced positive symptoms, negative symptoms, and improved functioning compared with placebo, but no significant effect was observed on depressive symptoms.

### Perinatal Depression

**Figure 6:**
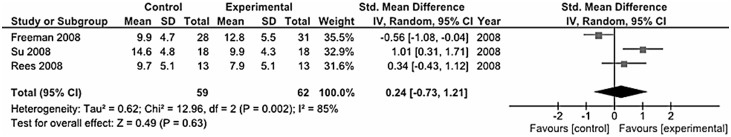
Forest plot showing individual and combined effect size estimates and 95% CIs for 3 trials conducted on pregnant women with major depressive disorder.

**Figure 7:**
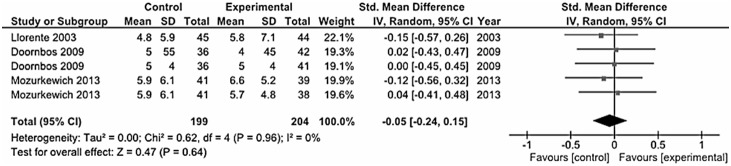
Forest plot showing individual and combined effect size estimates and 95% CIs for 3 trials conducted on healthy pregnant women for prevention of post-partum depression.

There were six trials aiming to explore the effects of omega-3 PUFA on perinatal depression. We distinguished between those studies conducted on pregnant women with MDD [82], [93], [99] (Figure 6) and those on apparently healthy women (primary prevention) [51], [68], [85] (Figure 7). However, both analyses led to inconclusive results (MDD in pregnancy, standardized mean difference – fixed effects-model: 0.08 SD, 95% CI: −0.29, 0.45; random-effects model: 0.24 SD, 95% CI: −0.73, 1.21; prevention of post-partum depression, standardized mean difference – fixed effects-model: 0.05 SD, 95% CI: −0.24, 0.15; random-effects model: −0.05 SD, 95% CI: −0.24, 0.15). Only one study [82] concluded that omega-3 fatty acids might have therapeutic benefits in depression during pregnancy. Besides the clinical efficacy of omega-3, in regard to the safety issue, it is important to underline that omega-3 fatty acids supplementation was well tolerated and no adverse effects were reported on the subjects treated and newborns in all studies.

### Depression as Secondary Outcome

**Figure 8:**
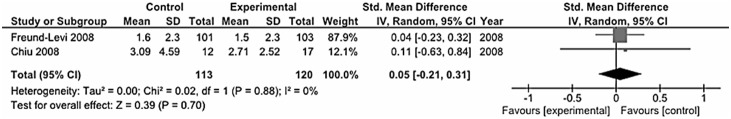
Forest plot showing individual and combined effect size estimates and 95% CIs for 2 trials conducted on patients with Alzheimer or mild cognitive impairment.

**Figure 9:**
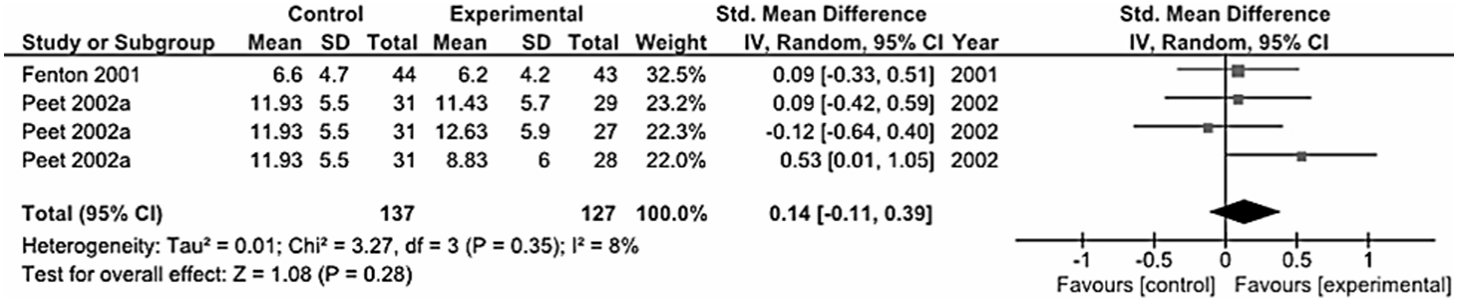
Forest plot showing individual and combined effect size estimates and 95% CIs for 2 trials conducted on patients with schizophrenia.

**Figure 10:**
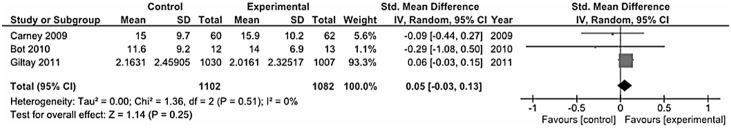
Forest plot showing individual and combined effect size estimates and 95% CIs for 3 trials conducted on patients with cardiovascular disease.

Among the trials conducted in patients with primary disease other than depression, those conducted on AD or mild cognitive impairment [50], [81] (Figure 8), schizophrenia [54], [90] (Figure 9), and CVDs [87], [94], [100] (Figure 10) reported inconclusive results, whereas the only study conducted on Parkinson’s disease patients in comorbidity with MDD [47], including those treated with antidepressants and those without, reported improvement in depressive symptoms and indicate that the intake of omega-3 PUFA can be used as adjuvant therapy in Parkinson’s disease patients. However, in one study conducted on schizophrenic patients with persistent ongoing symptoms [54], the authors reported a large placebo effect in patients on typical and new atypical antipsychotics and no difference was observed between active treatment and placebo, but in patients on clozapine, there was a clinically important and statistically significant effect of 2 g/day omega-3 PUFA treatment on the PANSS and its sub-scales.

### Depressive Symptoms in Healthy Subjects

**Figure 11:**
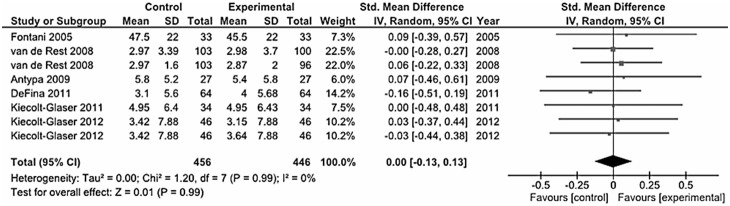
Forest plot showing individual and combined effect size estimates and 95% CIs for 6 trials conducted on healthy individuals for prevention of depressive symptoms.

The trials conducted on healthy subjects aimed to explore potential beneficial effects of omega-3 fatty acids as mood improving medicaments in the general population (Figure 11). Among the tot studies included [52], [66], [67], [88], [101], [102], the overall analysis showed a nearly null effect of this supplement on depressive symptoms in healthy subjects (standardized mean difference – fixed and random effects-model: 0.00 SD, 95% CI: −0.13, 0.13).

## Discussion

We demonstrated that the use of omega-3 PUFA as therapeutic agents was effective in patients with diagnosis of MDD and on depressive patients without a diagnosis of MDD, whereas inconclusive results were found for patients with other pathological conditions (namely schizophrenia and AD) as well as in healthy subjects and perinatal depression. The analysis of the studies on bipolar disorder showed a positive effect of the omega-3 PUFA, but the evidence is weakened due to the exclusion from the quantitative analysis of three studies that may affect the overall effect of the supplement. When the studies conducted on patients with MDD or those on patients with depressive symptoms but not rigorous evaluation by health professionals were pooled together, a general positive effect of omega-3 PUFA was found.

As previously reported [15], the studies that mostly negatively influenced the pooled results of the non-MDD patients included non-homogenous individuals, since their enrolment was in settings such as general practice surgeries, shopping malls, and university freshman fairs [86], newspaper, radio and television advertising, and flyers posted [70], and through a Community Mental Health Service, general practices, and advertisements in community newspapers [79]. Despite the idea of a widely available low cost supplement that could assist those being treated for a current depressive episode in a community setting is highly desirable, a lack of rigor in patients’ selection may lead to the inclusion of subjects with normal emotional states, eventually affecting the results and, thus, challenging the model’s credibility. It is noteworthy that negative results came out mostly from studies sharing this methodology [70], [79], [86]. Moreover, as reported by the authors [79], [86], both experimental and control groups improved significantly, usually indicative of a major placebo response which is expected to exert a meaningful clinical effect in the treatment of such “subthreshold” depressed subjects [103]. A recent meta-analysis demonstrated that the relative efficacy of the active drug compared to placebo in clinical trials for MDD is highly heterogeneous across studies, with a worse performance in showing a superiority of the drug *versus* placebo for studies with placebo response rates ≥30% [104]. Thus, the studies quality decreased when placebo response rates were not mantained below this critical threshold that may depend on the non-homogenous depressive “phenotypes” of the subjects enrolled. The non-MDD group also included four studies conducted on patients with depressive disorders despite ongoing antidepressant therapy [54], [69], [79], [97]. These results should be considered with caution, because these studies may include those “non-responder” subjects that generally fail to reach remission with the first anti-depressant therapy and have higher relapse rates and poorer outcomes than those who remit [105]. Studies conducted in this subgroup of patients can explain not clearly favorable effects of omega-3 PUFA on depressive symptoms in these studies and puzzling results.

Previous meta-analyses included all RCTs with little distinction among population groups, leading to controversial results, such as overall benefit [106], [107] and negligible effects [27], [28] of omega-3 PUFA against depressive symptoms, especially due to the high heterogeneity of studies. The following studies improved some methodological issues (i.e., better definition of inclusion criteria, especially in the distinction between the definition of MDD and other depressive disorders) and focused attention on specific aspects of omega-3 administration (i.e., dosage, EPA:DHA ratio) leading to the conclusion that administration of EPA, rather than DHA, is responsible for the beneficial effects of omega-3 PUFA intake as therapeutic agents in patients with depressive disorders [108], [109] and supplements containing EPA ≥60%, in dose range from 200 to 2200 mg EPA in excess of DHA, were effective against primary depression. On the contrary, the last meta-analytic study [14] reported small, non-significant benefit of omega-3 PUFA for the treatment of MDD, generally in contrast with the aforementioned previous meta-analyses, but some methodological issues in study selection have arisen [15], [16]. Taking into account that pathophysiological processes of depressive symptoms involved in MDD patients are likely to be very different from those in patients with depression occurring in other clinical conditions (i.e., bipolar disorder, pregnancy, primary diseases others than depression) and in non-homogenous patients (i.e., community sample of individuals), we used a different approach to analyze the RCTs using omega-3 PUFA supplementation against depressive symptoms, grouping the studies by type of diagnosis of depression and taking into account any possible health condition that may influence the onset of the depression as well as the response to therapy. Other meta-analyses reported that the more severe was the depression, the more likely omega-3 PUFA supplementation would reduce depressive symptoms. We failed to demonstrate such a result, and we consider this finding as a surrogate of our observation that, overall, the efficacy of omega-3 PUFA was mostly related to a specific DSM-based diagnosis of MDD. Hence, this latter has been translated in a correlation of efficacy to more severe symptoms whereas, according to our results, we hypothesized that this efficacy may be more related to the specific pathophysiological processes of the MDD rather than to its severity. Compared with previous meta-analyses, the differences of findings may depend on the additional number of RCTs published since the publication of the last study [37], [40], [53], [60], [61], [66], [68], [69], [84], [89], [92], [94]–[97], [100]–[102], the increasing number of participants which vary the overall weight of previous studies, the requirement for public registration of trials resulting in an increase of general studies’ quality and may be responsible for the decreased evidence of publication bias.

Since the pathophysiological mechanisms and the therapeutic approach for bipolar disorder differ from those of MDD [22], [23], when previous analyses included and pooled findings of studies conducted on these groups of different patients, they led to inconclusive results. It has been hypothesized that the efficacy of omega-3 PUFA may be different in the depressive phase rather than the maniacal episode [110], and recent systematic analysis of trials focused on this topic showed positive effects of omega-3 PUFA as an adjunctive treatment for depressive but not mania in bipolar disorder patients [111], [112]. Thus, we separately grouped the studies conducted on patients with bipolar disorder and explored efficacy of omega-3 PUFA in ameliorating the depressive symptoms, finding a significant efficacy of the supplement in two [65], [75] out of the three trials. Despite the positive results, it is noteworthy to underline that we had to exclude, due to missing of data, four studies [58], [59], [61], [63] conducted on bipolar patients reporting poor effect of the omega-3 PUFA intervention, thus weakening our findings. There is a need of well-designed, high quality studies, which may clarify the potential effects of omega-3 PUFA supplement in patients with rigorously diagnosed bipolar disorder.

Regarding the substantial inefficacy of the omega-3 PUFA in patients with primary diseases other than depression, it may be possible that these studies are more likely to suffer from publication bias, since depression was often a secondary outcome. Despite this methodological issue, the effects of the omega-3 PUFA may have been also affected by factors particularly related to the primary disease. Regarding the studies conducted on patients with CVDs, the analysis included very heterogeneous populations, namely patients with coronary heart disease [87], with diabetes mellitus [94], and post myocardial infarction [100], that may have been responsible for the inconclusive results. Moreover, it has been recently reported that supplementation of EPA in diabetes mellitus patients with comorbid MDD poorly affect biological risk factors for adverse outcome observed in this category of patients [113]. The RCTs conducted on patients with mild cognitive impairment or AD revealed poor efficacy of omega-3 PUFA in ameliorating the depressive symptoms. It has been reported that molecular mechanisms and pathways that underlie the pathogenesis of depression (i.e., impairment in the signaling of some neurotrophins such as Transforming-Growth-Factor-β1 and Brain-derived-neurotrophic-factor) are also involved in the pathogenesis of AD [114], [115], thus the omega-3 PUFA supplementation may not be the optimal pharmacological approach for this specific group of patients [116]–[118]. The two trials (including different dosages) conducted on schizophrenic patients with persistent ongoing symptoms resulted in limited effects of the omega-3 PUFA on patients’ affective states. These results may be attributable to some psychotic symptoms (i.e., negative symptoms) that may directly influence (i.e., improve) depression-rating scores. Moreover, these patients were receiving different types of antipsychotics such as first- and second-generation antipsychotics that may differently affect (positively or negatively) the final effects of omega-3 PUFA on depressive symptoms. Equally, we also reported that trials focused on perinatal depression demonstrated scarce efficacy of omega-3 on depressive symptoms. The supplemented omega-3 PUFA may have compensated the increased demand of the developing fetus during pregnancy and neonate during lactation rather than contributing to therapeutic efficacy by reducing depressive symptoms [119]. Finally, depression examined as secondary outcome could suffer by changing of the measurement depending on the improvement (or worsening) of the underlying primary disease.

Regarding the different efficacy of EPA compared with DHA and EPA-DHA combinations, the analysis of RCTs grouped according to type of omega-3 PUFA administered confirmed the findings of previous meta-analysis and substantial stronger pooled results of studies using EPA rather than DHA. However, as previously reported [109], the aforementioned methodological issues may have biased the results in favor of efficacy for EPA-containing preparations suggesting that the reported benefits on depressive symptoms in this group of studies may not therefore be definitively attributed only to the EPA content of the supplementation regimen and also that further studies are needed in this field. Whether EPA, rather than DHA, is effective in ameliorating depression in specific groups of patients, the different effects of these classes of omega-3 PUFA is a challenge to be explained convincingly, since DHA is a major structural component of neuronal membranes, and we can hypothesize that increasing its nutritional availability would have beneficial effects on brain function, rather than EPA, which is present at levels several hundred-fold lower [120]. Possible explanations of the beneficial role of EPA are the following: (i) the anti-inflammatory effects of EPA-derived eicosanoids [121] and its oxidized derivatives [122] (ii) its efficacy at reducing the inflammatory cytokines tumor necrosis factor-alpha (TNF-α), IL-6, and IL-1b [123] through inhibition of the activity of nuclear factor kappa-B (NF-kB) [124]; (iii) *in vivo* evidence of a more effective anti-inflammatory action of dietary EPA compared with DHA [125]. Moreover, DHA has been reported to be poorly incorporated in the human brain [126], and EPA may facilitate an increase in brain DHA levels after its conversion [127]. Finally, EPA supplementation has been associated with N-acetyl-aspartate increase in brain, a marker for neuronal homeostasis, suggesting its role as a neuroprotective agent [80]. Together with the inflammation theory of depression [8], chronic intake of omega-3 fatty acids has been reported to play an important role in neuronal structure and function [128]. However, such hypotheses are not completely exhaustive and further research is needed to better identify the specific molecular mechanisms underlying clinical efficacy of omega-3 PUFA (both EPA and DHA) in preventing or ameliorating depression.

The studies excluded from this systematic review were not comparable in terms of methodology used, and their exclusion was needed in order to reduce differences among RCTs and improve data quality (i.e., reduce selection bias). On the other hand, these trials may still be directly relevant to the topic of the present study, and a specific discussion (e-discussion) may strengthen conclusion retrieved from this meta-analysis. Moreover, we discussed in a specific section of the e-discussion about the studies quality and potential sources of heterogeneity.

The main limitation of this study was the inability to control all the many potential sources of heterogeneity. Despite the fact that a logical grouping of trials was performed, a non-modifiable degree of heterogeneity, due to specific characteristics of all trials included, still weakened the pooled analysis of these studies. However, compared with older studies, the inclusion of the updated RCTs strengthened the conclusions of the effects of omega-3 PUFA intake on depressive disorders.

To sum up, trials conducted in individuals with a diagnosis of MDD provided evidence that omega-3 PUFA supplementation has beneficial clinical effects on depressive status. Evidence of their efficacy was provided also for patients with bipolar disorder, whereas no evidence was found for individuals included in the other diagnostic groups. According to our findings, in RCTs with omega-3 PUFA supplementation in healthy subjects and patients with schizophrenia, AD and CVD seems to result ineffective.

## Supporting Information

Checklist S1 **PRISMA checklist for meta-analyses studies.**
(DOC)

Discussion S1 **Additional discussion.**
(DOCX)
