# Supplementary material for: Role of Omega-3 Fatty Acids in the Treatment of Depressive Disorders: A Comprehensive Meta-Analysis of Randomized Clinical Trials
Source: PLoS One. 2014 May 7;9(5):e96905. doi: 10.1371/journal.pone.0096905 (PMC4013121; doi:10.1371/journal.pone.0096905)
Supplement: Discussion S1 — Additional discussion. (DOCX) [file pone.0096905.s002.docx]

**Discussion S1**

The significant heterogeneity among the studies included in this meta-analysis may be related to potential limitations of the trials depending on their quality. Indeed, 11 studies [[1-11](#_ENREF_1)] did not report blinding process, 3 studies [[5](#_ENREF_5), [12](#_ENREF_12), [13](#_ENREF_13)] did not report nature of placebo and 3 [[3](#_ENREF_3), [14](#_ENREF_14), [15](#_ENREF_15)] did not report number dropouts. In 16 studies [[1](#_ENREF_1), [2](#_ENREF_2), [4](#_ENREF_4), [9](#_ENREF_9), [11](#_ENREF_11), [12](#_ENREF_12), [16-25](#_ENREF_16)] baseline characteristics were not clearly reported and in one study [[5](#_ENREF_5)] significantly differed between intervention and placebo groups. Thirty-one studies [[1](#_ENREF_1), [4-6](#_ENREF_4), [9](#_ENREF_9), [12](#_ENREF_12), [14-17](#_ENREF_14), [19](#_ENREF_19), [20](#_ENREF_20), [22-24](#_ENREF_22), [26-40](#_ENREF_26)] did not mention to have controlled for dietary intake of fish or omega-3 fatty acids and 18 [[1](#_ENREF_1), [4](#_ENREF_4), [6](#_ENREF_6), [8-10](#_ENREF_8), [12](#_ENREF_12), [21](#_ENREF_21), [26](#_ENREF_26), [27](#_ENREF_27), [29](#_ENREF_29), [30](#_ENREF_30), [32](#_ENREF_32), [34](#_ENREF_34), [35](#_ENREF_35), [37](#_ENREF_37), [41](#_ENREF_41), [42](#_ENREF_42)] did not assess for compliance using red blood cells membrane or plasma phospholipid analyses. The quality of some studies was affected by dropouts, ranging from 1% [[21](#_ENREF_21)] to 30% [[43](#_ENREF_43)] of the study participants, whereas nearly half of the studies did not use an intention-to-treat design. Another potential source of heterogeneity among trials was the lack of results by gender. Due to the different prevalence rates of depression between men and women and different metabolic pathways of omega-3 PUFA (such as higher levels of plasma DHA in women than in men despite identical diet) [[44](#_ENREF_44)], it would be highly desirable to stratify results by gender.

Among the studies excluded from the systematic review, studies reporting the depressive status as a categorical variable were conducted for the prevention of post-partum depression [[14](#_ENREF_14), [45](#_ENREF_45), [46](#_ENREF_46)] and on women with bipolar disorder who discontinued all conventional psychotropic medications while attempting to conceive [[26](#_ENREF_26)]. All of these studies showed comparable outcomes between intervention and control groups, similarly to the RCTs included in the meta-analysis conducted on analogous population. However, one study [[14](#_ENREF_14)] reported that women with BDI scores >10 showed a significant improvement in their mood scores after pregnancy, despite still having more depressive symptoms (higher mood scores) in the post-partum than the women with no depressive symptoms at 20 weeks. Another study conducted on CVD survivors categorizing the outcome (depression yes/not) reported no beneficial effects of a long-term, low-dose supplementation of omega-3 fatty acids on depressive symptoms [[1](#_ENREF_1)]. In two studies [[47](#_ENREF_47), [48](#_ENREF_48)] on men with angina and healthy subjects, respectively, participants were advised to eat fish (>3 servings per week) with no effects on mood and depression scores. One trial with poorly comparable placebo, used omega-3 18:12 fish oil (containing 18% EPA and 12% DHA) *vs.* commercial krill oil (containing itself unquantifiable omega-3 fatty acids) administered on women with premenstrual syndrome [[41](#_ENREF_41)], a study using Efamol in women with severe premenstrual syndrome [[49](#_ENREF_49)], and a trial not placebo-controlled conducted on adolescent with juvenile bipolar disorder [[50](#_ENREF_50)], reported all beneficial effect of omega-3 fatty acids on depressive symptoms. Among the studies that used poorly comparable scores to assess symptoms severity of depression, one conducted on women with premenstrual syndrome used a visual analogue score [[27](#_ENREF_27)] and one conducted on subjects with post viral fatigue measured depression on a scale of from 0-3 (0, absent; 1, mild; 2, moderate; 3, severe) [[16](#_ENREF_16)], both reporting a decrease of the mean severity of depressive symptoms in the intervention group supplemented with omega-3 fatty acids. In conclusion, despite a significant heterogeneity in study design and quality, the excluded trials reported comparable results to those trials included in the systematic review conducted in the analogue population.

**E-discussion references**

[1] Andreeva VA, Galan P, Torres M, Julia C, Hercberg S, Kesse-Guyot E. Supplementation with B vitamins or n-3 fatty acids and depressive symptoms in cardiovascular disease survivors: ancillary findings from the SUpplementation with FOLate, vitamins B-6 and B-12 and/or OMega-3 fatty acids (SU.FOL.OM3) randomized trial. Am J Clin Nutr 2012;96:208-14.

[2] B. D, S.A. VG, D.A. D-B, A. S, J. K, F.A. M. Supplementation of a low dose of DHA or DHA+AA does not prevent peripartum depressive symptoms in a small population based sample. Prog Neuropsychopharmacol Biol Psychiatry 2009;33:49-52.

[3] Fontani G, Corradeschi F, Felici A, Alfatti F, Bugarini R, Fiaschi AI, et al. Blood profiles, body fat and mood state in healthy subjects on different diets supplemented with Omega-3 polyunsaturated fatty acids. Eur J Clin Invest 2005;35:499-507.

[4] Peet M, Horrobin DF. A dose-ranging study of the effects of ethyl-eicosapentaenoate in patients with ongoing depression despite apparently adequate treatment with standard drugs. Arch Gen Psychiatry 2002;59:913-9.

[5] Marangell LB, Martinez JM, Zboyan HA, Kertz B, Kim HF, Puryear LJ. A double-blind, placebo-controlled study of the omega-3 fatty acid docosahexaenoic acid in the treatment of major depression. Am J Psychiatry 2003;160:996-8.

[6] Su KP, Huang SY, Chiu CC, Shen WW. Omega-3 fatty acids in major depressive disorder. A preliminary double-blind, placebo-controlled trial. Eur Neuropsychopharmacol 2003;13:267-71.

[7] Gertsik L, Poland RE, Bresee C, Rapaport MH. Omega-3 fatty acid augmentation of citalopram treatment for patients with major depressive disorder. J Clin Psychopharmacol 2012;32:61-4.

[8] Zanarini MC, Frankenburg FR. omega-3 Fatty acid treatment of women with borderline personality disorder: a double-blind, placebo-controlled pilot study. Am J Psychiatry 2003;160:167-9.

[9] Nemets H, Nemets B, Apter A, Bracha Z, Belmaker RH. Omega-3 treatment of childhood depression: a controlled, double-blind pilot study. Am J Psychiatry 2006;163:1098-100.

[10] Freeman MP, Davis M, Sinha P, Wisner KL, Hibbeln JR, Gelenberg AJ. Omega-3 fatty acids and supportive psychotherapy for perinatal depression: a randomized placebo-controlled study. J Affect Disord 2008;110:142-8.

[11] DeFina LF, Marcoux LG, Devers SM, Cleaver JP, Willis BL. Effects of omega-3 supplementation in combination with diet and exercise on weight loss and body composition. Am J Clin Nutr 2011;93:455-62.

[12] Nemets B, Stahl Z, Belmaker RH. Addition of omega-3 fatty acid to maintenance medication treatment for recurrent unipolar depressive disorder. Am J Psychiatry 2002;159:477-9.

[13] Llorente AM, Jensen CL, Voigt RG, Fraley JK, Berretta MC, Heird WC. Effect of maternal docosahexaenoic acid supplementation on postpartum depression and information processing. Am J Obstet Gynecol 2003;188:1348-53.

[14] Mattes E, McCarthy S, Gong G, van Eekelen JA, Dunstan J, Foster J, et al. Maternal mood scores in mid-pregnancy are related to aspects of neonatal immune function. Brain Behav Immun 2009;23:380-8.

[15] Frangou S, Lewis M, Wollard J, Simmons A. Preliminary in vivo evidence of increased N-acetyl-aspartate following eicosapentanoic acid treatment in patients with bipolar disorder. J Psychopharmacol 2007;21:435-9.

[16] Behan PO, Behan WM, Horrobin D. Effect of high doses of essential fatty acids on the postviral fatigue syndrome. Acta Neurol Scand 1990;82:209-16.

[17] Freund-Levi Y, Basun H, Cederholm T, Faxen-Irving G, Garlind A, Grut M, et al. Omega-3 supplementation in mild to moderate Alzheimer's disease: effects on neuropsychiatric symptoms. Int J Geriatr Psychiatry 2008;23:161-9.

[18] Rondanelli M, Giacosa A, Opizzi A, Pelucchi C, La Vecchia C, Montorfano G, et al. Long chain omega 3 polyunsaturated fatty acids supplementation in the treatment of elderly depression: effects on depressive symptoms, on phospholipids fatty acids profile and on health-related quality of life. J Nutr Health Aging 2011;15:37-44.

[19] Hirashima F, Parow AM, Stoll AL, Demopulos CM, Damico KE, Rohan ML, et al. Omega-3 fatty acid treatment and T(2) whole brain relaxation times in bipolar disorder. Am J Psychiatry 2004;161:1922-4.

[20] Silvers KM, Woolley CC, Hamilton FC, Watts PM, Watson RA. Randomised double-blind placebo-controlled trial of fish oil in the treatment of depression. Prostaglandins Leukot Essent Fatty Acids 2005;72:211-8.

[21] Antypa N, Smelt AH, Strengholt A, Van der Does AJ. Effects of omega-3 fatty acid supplementation on mood and emotional information processing in recovered depressed individuals. J Psychopharmacol 2012;26:738-43.

[22] Amminger GP, Schafer MR, Papageorgiou K, Klier CM, Cotton SM, Harrigan SM, et al. Long-chain omega-3 fatty acids for indicated prevention of psychotic disorders: a randomized, placebo-controlled trial. Arch Gen Psychiatry 2010;67:146-54.

[23] Rondanelli M, Giacosa A, Opizzi A, Pelucchi C, La Vecchia C, Montorfano G, et al. Effect of omega-3 fatty acids supplementation on depressive symptoms and on health-related quality of life in the treatment of elderly women with depression: a double-blind, placebo-controlled, randomized clinical trial. J Am Coll Nutr 2010;29:55-64.

[24] Rizzo AM, Corsetto PA, Montorfano G, Opizzi A, Faliva M, Giacosa A, et al. Comparison between the AA/EPA ratio in depressed and non depressed elderly females: omega-3 fatty acid supplementation correlates with improved symptoms but does not change immunological parameters. Nutr J 2012;11:82.

[25] Kiecolt-Glaser JK, Belury MA, Andridge R, Malarkey WB, Glaser R. Omega-3 supplementation lowers inflammation and anxiety in medical students: a randomized controlled trial. Brain Behav Immun 2011;25:1725-34.

[26] Marangell LB, Suppes T, Ketter TA, Dennehy EB, Zboyan H, Kertz B, et al. Omega-3 fatty acids in bipolar disorder: clinical and research considerations. Prostaglandins Leukot Essent Fatty Acids 2006;75:315-21.

[27] Sohrabi N, Kashanian M, Ghafoori SS, Malakouti SK. Evaluation of the effect of omega-3 fatty acids in the treatment of premenstrual syndrome: "a pilot trial". Complement Ther Med 2013;21:141-6.

[28] Grenyer BF, Crowe T, Meyer B, Owen AJ, Grigonis-Deane EM, Caputi P, et al. Fish oil supplementation in the treatment of major depression: a randomised double-blind placebo-controlled trial. Prog Neuropsychopharmacol Biol Psychiatry 2007;31:1393-6.

[29] Jazayeri S, Tehrani-Doost M, Keshavarz SA, Hosseini M, Djazayery A, Amini H, et al. Comparison of therapeutic effects of omega-3 fatty acid eicosapentaenoic acid and fluoxetine, separately and in combination, in major depressive disorder. Aust N Z J Psychiatry 2008;42:192-8.

[30] Hallahan B, Hibbeln JR, Davis JM, Garland MR. Omega-3 fatty acid supplementation in patients with recurrent self-harm. Single-centre double-blind randomised controlled trial. Br J Psychiatry 2007;190:118-22.

[31] Warren G, McKendrick M, Peet M. The role of essential fatty acids in chronic fatigue syndrome. A case-controlled study of red-cell membrane essential fatty acids (EFA) and a placebo-controlled treatment study with high dose of EFA. Acta Neurol Scand 1999;99:112-6.

[32] Keck PE, Jr., Mintz J, McElroy SL, Freeman MP, Suppes T, Frye MA, et al. Double-blind, randomized, placebo-controlled trials of ethyl-eicosapentanoate in the treatment of bipolar depression and rapid cycling bipolar disorder. Biol Psychiatry 2006;60:1020-2.

[33] Gracious BL, Chirieac MC, Costescu S, Finucane TL, Youngstrom EA, Hibbeln JR. Randomized, placebo-controlled trial of flax oil in pediatric bipolar disorder. Bipolar Disord 2010;12:142-54.

[34] Chiu CC, Huang SY, Chen CC, Su KP. Omega-3 fatty acids are more beneficial in the depressive phase than in the manic phase in patients with bipolar I disorder. J Clin Psychiatry 2005;66:1613-4.

[35] Frangou S, Lewis M, McCrone P. Efficacy of ethyl-eicosapentaenoic acid in bipolar depression: randomised double-blind placebo-controlled study. Br J Psychiatry 2006;188:46-50.

[36] Mozurkewich EL, Clinton CM, Chilimigras JL, Hamilton SE, Allbaugh LJ, Berman DR, et al. The Mothers, Omega-3, and Mental Health Study: a double-blind, randomized controlled trial. Am J Obstet Gynecol 2013;208:313 e1-9.

[37] Stoll AL, Severus WE, Freeman MP, Rueter S, Zboyan HA, Diamond E, et al. Omega 3 fatty acids in bipolar disorder: a preliminary double-blind, placebo-controlled trial. Arch Gen Psychiatry 1999;56:407-12.

[38] Chiu CC, Su KP, Cheng TC, Liu HC, Chang CJ, Dewey ME, et al. The effects of omega-3 fatty acids monotherapy in Alzheimer's disease and mild cognitive impairment: a preliminary randomized double-blind placebo-controlled study. Prog Neuropsychopharmacol Biol Psychiatry 2008;32:1538-44.

[39] Carney RM, Freedland KE, Rubin EH, Rich MW, Steinmeyer BC, Harris WS. Omega-3 augmentation of sertraline in treatment of depression in patients with coronary heart disease: a randomized controlled trial. JAMA 2009;302:1651-7.

[40] Giltay EJ, Geleijnse JM, Kromhout D. Effects of n-3 fatty acids on depressive symptoms and dispositional optimism after myocardial infarction. Am J Clin Nutr 2011;94:1442-50.

[41] Sampalis F, Bunea R, Pelland MF, Kowalski O, Duguet N, Dupuis S. Evaluation of the effects of Neptune Krill Oil on the management of premenstrual syndrome and dysmenorrhea. Altern Med Rev 2003;8:171-9.

[42] Fux M, Benjamin J, Nemets B. A placebo-controlled cross-over trial of adjunctive EPA in OCD. J Psychiatr Res 2004;38:323-5.

[43] Mischoulon D, Papakostas GI, Dording CM, Farabaugh AH, Sonawalla SB, Agoston AM, et al. A double-blind, randomized controlled trial of ethyl-eicosapentaenoate for major depressive disorder. J Clin Psychiatry 2009;70:1636-44.

[44] Giltay EJ, Gooren LJ, Toorians AW, Katan MB, Zock PL. Docosahexaenoic acid concentrations are higher in women than in men because of estrogenic effects. Am J Clin Nutr 2004;80:1167-74.

[45] Krauss-Etschmann S, Shadid R, Campoy C, Hoster E, Demmelmair H, Jimenez M, et al. Effects of fish-oil and folate supplementation of pregnant women on maternal and fetal plasma concentrations of docosahexaenoic acid and eicosapentaenoic acid: a European randomized multicenter trial. Am J Clin Nutr 2007;85:1392-400.

[46] Makrides M, Gibson RA, McPhee AJ, Yelland L, Quinlivan J. Effect of DHA supplementation during pregnancy on maternal depression and neurodevelopment of young children: a randomized controlled trial. JAMA 2010;304:1675-83.

[47] Ness AR, Gallacher JE, Bennett PD, Gunnell DJ, Rogers PJ, Kessler D, et al. Advice to eat fish and mood: a randomised controlled trial in men with angina. Nutr Neurosci 2003;6:63-5.

[48] Beezhold BL, Johnston CS. Restriction of meat, fish, and poultry in omnivores improves mood: a pilot randomized controlled trial. Nutr J 2012;11:9.

[49] Puolakka J, Makarainen L, Viinikka L, Ylikorkala O. Biochemical and clinical effects of treating the premenstrual syndrome with prostaglandin synthesis precursors. J Reprod Med 1985;30:149-53.

[50] Clayton EH, Hanstock TL, Hirneth SJ, Kable CJ, Garg ML, Hazell PL. Reduced mania and depression in juvenile bipolar disorder associated with long-chain omega-3 polyunsaturated fatty acid supplementation. Eur J Clin Nutr 2009;63:1037-40.
